# Identifying levels of general distress in first line mental health services: can GP- and eHealth clients’ scores be meaningfully compared?

**DOI:** 10.1186/s12888-017-1552-3

**Published:** 2017-12-01

**Authors:** Jan van Bebber, Johanna T. W. Wigman, Lex Wunderink, Jorge N. Tendeiro, Marieke Wichers, Janneke Broeksteeg, Bart Schrieken, Sjoerd Sytema, Berend Terluin, Rob R. Meijer

**Affiliations:** 1 University Medical Center Groningen, Interdisciplinary Center Psychopathology and Emotion Regulation (ICPE), University of Groningen, P.O. Box 30.001, 9700 RB Groningen, The Netherlands; 2 Department of Education and Research, GGZ Friesland, Leeuwarden, The Netherlands; 3 University Medical Center Groningen, Rob Giel Research Center (RGOc), University of Groningen, Groningen, The Netherlands; 4 Department of Psychometrics and Statistics, University of Groningen, Groningen, The Netherlands; 5 Interapy, Amsterdam, The Netherlands; 6 Department of General Practice and Elderly Care Medicine, EMGO Institute for Health and Care Research, VU University Medical Center, Amsterdam, The Netherlands

## Abstract

**Background:**

The Four-Dimensional Symptom Questionnaire (4DSQ) (Huisarts Wetenschap 39: 538–47, 1996) is a self-report questionnaire developed in the Netherlands to distinguish non-specific general distress from depression, anxiety, and somatization. This questionnaire is often used in different populations and settings and there is a paper-and-pencil and computerized version.

**Methods:**

We used item response theory to investigate whether the 4DSQ measures the same construct (structural equivalence) in the same way (scalar equivalence) in two samples comprised of primary mental health care attendees: (i) clients who visited their General Practitioner responded to the 4DSQ paper-and-pencil version, and (ii) eHealth clients responded to the 4DSQ computerized version. Specifically, we investigated whether the distress items functioned differently in eHealth clients compared to General Practitioners’ clients and whether these differences lead to substantial differences at scale level.

**Results:**

Results showed that in general structural equivalence holds for the distress scale. This means that the distress scale measures the same construct in both General Practitioners’ clients and eHealth clients. Furthermore, although eHealth clients have higher observed distress scores than General Practitioners’ clients, application of a multiple group generalized partial credit response model suggests that scalar equivalence holds.

**Conclusions:**

The same cutoff scores can be used for classifying respondents as having low, moderate and high levels of distress in both settings.

**Electronic supplementary material:**

The online version of this article (10.1186/s12888-017-1552-3) contains supplementary material, which is available to authorized users.

## Background

In many European countries, including the Netherlands, consulting a General Practitioner (GP) is a formal prerequisite for referral to specialized care providers in case of mental health problems. As such, GPs fulfill the role of gatekeeper for mental health services and with this task comes the need for adequate and efficient methods to screen for possible mental health problems. Many tools such as structured interviews and questionnaires have been developed to facilitate this process, and the latter are also incorporated in assessment batteries of various eHealth providers. The 4DSQ [1] is such a questionnaire. The 4DSQ is a self-report questionnaire developed in the Netherlands to distinguish non-specific general distress from depression, anxiety, and somatization.

As with many questionnaires, the 4DSQ is often administered in various *populations* in different *settings* and with different *mediums*: A test or questionnaire may be designed for implementation in, for example, the general population, the working population, or the population of ambulant health care recipients. With *setting*, we refer to the specific situation in which the questionnaire is applied (e.g., outpatient clinic or hospital). With *medium*, we refer to the way data are collected (e.g., experiments or structured interviews). Note that a test or questionnaire applied in practice always has a specific combination of these three factors. To keep things simple, we will use the term ***application mode*** for the specific combination of these three factors in the remainder of this introduction.

What can we learn from the literature regarding equivalence of application modes? With regard to medium effects (paper and pencil versus computerized), perhaps the most important lesson is that different ‘research designs’ lead to different conclusions. Where the study design is experimental, data appear to be equivalent in terms of factorial structure, reliability, means and standard deviations [2]. When data are collected by different mediums in applied settings though, especially core coefficients of score distributions diverge. That is, significant and relevant differences in central tendency and spread appear between both conditions due to, for example, differential social-desirability responding combined with effects of differences in demographic backgrounds of respondents between data collection frames [3]. In many clinical settings data are not collected anonymously, and data are collected using different mediums and from various populations. In all of these cases, there is a great need for information about whether the test or questionnaire assesses the same construct across application modes. This property has been labeled *structural equivalence* [4, 5]*.*

Furthermore, it is important to verify whether scale scores have the same meaning across application modes. This property is referred to as *scalar* equivalence. That is, equal scale scores should reflect the same levels of the underlying trait in various application modes. This is because scalar equivalence is a prerequisite for meaningful score comparisons *across* application modes and thus also for justifying the usage of, for example, the same cutoff scores for classification of respondents. The framework of Item Response Theory (IRT) is very appealing because of its equivalence property [6]. That is, differences in item functioning may be characterized in a way that is not affected by differences in the trait distributions between application modes.

In the research discussed in this paper, both samples consisted of individuals who seek help- and, or assistance from primary mental health care providers. The setting was an intake procedure at General Practioner practices for the first sample, and an intake procedure of an eHealth provider for the second sample. The medium was a paper-and-pencil administration for the first sample, and a computerized administration for the second. Note that the eHealth setting implied online testing. We refer to the first sample as the GP sample, and to the second sample as the eHealth sample in the remainder of this article.

### Aims of this study

In this study, we compared the psychometric properties of the 4DSQ distress scale in two samples of which the application modes differed with respect to the factors that have been explained above. More specifically, we examined whether

(i) the distributions of total scores differed between samples in terms of central tendency and spread;
(ii) a suitable IRT model would fit the data;
(iii) the distress items functioned similarly in both samples (structural equivalence);
(iv) equal total scores reflected the same levels of distress in both groups (scalar equivalence);
(v) the two samples differ in their distribution of latent scores, and
(vi) measurement precision differs between samples and along the latent distress continuum.

## Methods

### The Four-Dimensional Symptom Questionnaire (4DSQ): background information and existing research

The 4DSQ is a self-report questionnaire that can be used to distinguish non-specific general distress (16 items) from depression (6 items), anxiety (12 items), and somatization (16 items). Although initially developed for primary care settings, its validity has also been demonstrated in working populations [7] and in ambulant mental health services [8]. Respondents have to indicate the frequency of specific symptom experiences during the past week on a five-point scale (‘Not present’, ‘Sometimes’, ‘Regularly’, ‘Often’, and ‘Constantly present’). In practice, the three highest item scores (2–4) are recoded into a 2-score to avoid response bias caused by extreme responding [1]. Recoded item scores are summed for each scale. The total score for the distress scale thus ranges from 0 to 32. In practice [1], scores lower than 11 are interpreted as representing *low* levels of distress, scores in between 11 and 20 represent *moderate* levels of distress, and scores larger than 20 represent *high* levels of distress. These cutoff values are based on clinical experience and expertise; that is, observations that were made by clinicians in a non-systematic way (Terluin, 2016; personal communication). Note that the same cut-off scores for classifying respondents as having low, moderate, and high levels of distress are used in each application mode, though it has not yet been shown that scalar equivalence holds between application modes. Thus, evidence that justifies the use of the same cut-off scores across application modes is strongly needed.

Terluin [9] found that the scores on the four scales can be described adequately by unidimensional (common) factor models, and all four scales were found to be invariant with respect to gender, age and educational level of respondents [10]. Furthermore, the model with four factors showed a better fit than alternative models where, for example, the items of the depression scale were allowed to load on two separate factors [9].

Professionals applying the 4DSQ find the distress scale most informative, and compared to the other subscales of the instrument, it shows the strongest associations with various mental health indicators (see next paragraph). This makes the distress scale most often used in practice. Therefore, the focus of this study was to further investigate the psychometric characteristics of this scale. Terluin [7, 8] found that the reliability of the distress scale (coefficient alpha) was approximately .90 for both primary care clients and outpatients of mental health providers.

The structure of the nomological network was in accordance with the theoretical expectations: the distress scale correlated positively with other nonspecific measures of distress like the General Health Questionnaire (*r* = .58) and the Maastricht Questionnaire (*r* = .46), showing good convergent content validity. One frequently stated criticism is that the divergent content validity of the scale is relatively weak, because the distress scale also correlated highly with various measures of depression and anxiety, including the other 4DSQ subscales [9]. However, this is a common phenomenon for measures of distress, depression, and anxiety [10, 11]. Furthermore, regarding predictive validity, moderate positive associations with stress-related measures such as life events (*R* ^2^ = 11%) and psychosocial problems (*R* ^2^ = 30%) were found, with personality traits as Neuroticism (*R* ^2^ = 45%) and Mastery (*R* ^2^ = 29%), and also moderate negative relationships with indicators of social (*R* ^2^ = 31%) and occupational functioning (*R* ^2^ = 29%) were found [9].

### Participants

In the current study, we used datasets that have been collected years ago. 1142 clients who visited their GP in the Netherlands between 2004 and 2011 with need for mental health care were asked to fill out the paper-and-pencil version of the 4DSQ at their GPs’ practices. We selected those 1017 clients who filled out the questionnaire without omitting any item of the distress scale for further analysis. Mean age was 40.2 (SD = 14.9, age range 11–85 years), and 63.3% were female.

The eHealth sample comprised 1409 clients who contacted the Dutch eHealth provider Interapy^1^ in 2015 with need for mental health care. These individuals completed the intake procedure that included the online 4DSQ. Mean age in this sample was 35.7 (SD = 13.5, range 12–90), and 73.5% were female.

### The generalized partial credit model (GPCM)

To analyze the data, we used the GPCM [12]. The GPCM is an IRT model for polytomous items. In IRT, item categories (or boundaries between item categories) and persons are placed on a common latent scale (often denoted by θ). This latent scale represents a continuous construct, for example, depression. The distribution of persons on this latent scale may be conceived as approximately standardized. An IRT-model specifies the way in which characteristics of items and respondents influence (changes in) expected item scores of respondents. The GPCM is a generalization of the Rasch model [13] to polytomous items. Each item with k response categories is characterized by a discrimination parameter (*a*) and a set of k-1 interception parameters. The category interception parameters denote the locations on the latent trait at which the probability of endorsing the two corresponding response categories is equal. The discrimination parameter expresses how fast expected item scores change when differences between person parameter and item category interception parameters increase. Contrary to the Rasch model, in the GPCM items are allowed to differ in discrimination. The interested reader is referred to the Additional file 1 for more technical information on the GPCM.

The GPCM is based on the related assumptions of unidimensionality and local stochastic independence (LSI; antonym is Local Dependence, LD). Unidimensionality implies that the item scores can be explained by a dominant single latent variable (in this case distress) and LSI implies that the item scores are (essentially) uncorrelated when controlling for this latent variable. Before an IRT model is applied to empirical data, these assumptions should be checked. For more details on IRT, see [6, 14].

### Differential item functioning (DIF) and multiple group IRT analysis (MGIRT)

The relationship between trait level and expected item scores may differ between groups. In the context of IRT, this phenomenon is referred to as Differential Item Functioning (DIF). When exploring DIF in clinical scales, one may investigate (i) whether specific symptoms are more important (i.e., are more differentiating) for assessing a psychopathological domain in one group than in the other, and (ii) whether specific symptoms become manifest at different levels of psychopathology between groups. DIF of the first kind would result in different discrimination parameters between groups and DIF of the second kind would result in different interception parameters between groups. For the interested reader, the technical details of this procedure are given in the Additional file 1.

When item parameters differ between groups, expected item scores of respondents with equal trait levels that belong to different groups differ. The accumulation of these effects at the scale level may lead to differential test functioning (DTF). In this case, equal total scores of respondents between groups may actually reflect different (latent) trait levels. The relationship between total scale score metric and latent trait metric is expressed by the so-called Test Characteristic Curves (TCC). When these curves differ substantially between groups, comparisons of individual scores across groups should not be based on total scale scores but on latent trait levels. Consequently, using the total scale score metric in that case would not be appropriate for defining equal cutoff scores for respondents of both groups.

Multiple group IRT-analysis (MGIRT) offers the possibility to use data from multiple groups for deriving item parameter estimates, while model-fit is still assessed for each group separately. Increasing sample size leads to more precise item parameter estimates. All items and all persons may be placed on a common latent scale, anchoring the scale by using the theta distribution in the reference group. Furthermore, in case of more than two non-overlapping groups, differential item functioning can be assessed for each subgroup (or ‘focal groups’) relative to a chosen reference group.

When some items function differently between groups, it can be investigated whether DIF-effects cancel out (or are negligible) at the scale level as expressed by equal (or nearly equal) TCCs across groups. Even when this is not the case, latent distributions can be used for meaningful group comparisons, because these are based on collections of items that do not exhibit DIF with respect to the groups compared.

### MGIRT analyses

All IRT analyses were performed on the recoded (0–2) item scores, because these are used in practice. First, structural equivalence between the two samples (i.e. GP and eHealth clients) was investigated. To do this, we first conducted a multiple group analysis where item parameters were constrained to be equal across samples. In order to identify the latent distress continuum, we decided to restrict the mean theta-value of GP clients to equal zero and the standard deviation of theta values to equal one. The mean and standard deviation of theta-values in the sample of eHealth clients were computed using this restriction in combination with the item parameters estimated. We investigated model fit in both groups separately for each item, and inspected DIF effects across samples. Because the test statistics used for both assessing model fit and assessing DIF effects are very sensitive with large samples, we inspected the differences between observed and expected category score frequencies for different score levels (i.e., the total score without the item targeted) for those items that showed the worst fit (*p* <. 01). Instead of doing this for each score level, we collapsed score levels in such a way as to create expected category score frequencies of at least one hundred persons in each cell. Additionally, local independence between all item pairs was investigated. The interested reader is again referred to the Additional file 1 for technical details.

Second, in case some items would function differently across groups, we examined scalar equivalence by comparing the TCCs for both groups (based on the augmented model in which some items have group-specific parameter values). Additionally, we compared the latent distress distributions between groups in terms of central tendency (means) and spread (standard deviations).

Third, measurement precision, a local concept within the framework of IRT, was compared between groups. The information that individual items and sets of items provide depends on (i) the discriminative power of the items, and, (ii) the position (θ-value) of respondents on the latent scale. The closer the positions of respondent and item are on the latent continuum, the more information an item will provide for this specific respondent. With respect to distress, this reflects how well the intensity-levels of symptoms match clients’ levels of distress. The more information items provide, the lower the measurement error for individual distress scores. How much information an item provides along the latent scale is expressed by Item Information Functions (IIFs), and these functions may be summed to Test Information Functions (TIFs). These express how much information is provided at the scale level. Standard errors that are conditional on the latent trait level are simple inverse functions of the TIFs.

In order to investigate structural equivalence, we could also have used the well-known technique of multigroup confirmatory factor analysis. Note that this technique could not have been used to investigate the property of scalar equivalence, because with factor analytic techniques, differences in item means between groups are typically ignored by standardizing items scores prior to analysis. Furthermore, because measurement precision is assumed to be a global concept in the context of factor analysis, we would not have been able to investigate whether measurement precision varies along the latent distress continuum.

We used IRTPRO, version 3 [15] for deriving item- and person parameter estimates in the MGIRT, for performing the DIF-analyses, and for generating the TCCs and TIFs for both groups.

## Results

### Sample Descriptives for both groups

The means, standard deviations, and resulting standardized difference on the 4DSQ distress scale in both groups are displayed in Table 1. EHealth clients scored significantly (*F* = 136.09, *p* < .01) higher than GP clients and the spread of the scale scores was lower for eHealth clients than for GP attendees.

**Table 1:** Descriptive statistics 4DSQ distress scale and frequencies of category scores within the samples

	GP attendees		EHealth clients		D^a^
	Mean	SD	Mean	SD	
Distress	19.76	8.86	23.47	6.79	−.48
Low distress (S_x_ ≤ 10)	19.8%		5.3%		
Moderate distress (11 ≤ S_x_ ≤ 20)	27.6%		24.6%		
High distress (S_x_ ≥ 21)	52.6%		70.2%		

^a^ Standardized difference;

The percentage of clients that reported moderate levels of distress was comparable between groups. However, GP clients’ levels of distress fall much more often in the lowest category, whereas eHealth clients’ levels of distress fall much more often in the highest category.

### IRT-analyses: GP clients

As discussed in the methods section, the principle of LSI is crucial for justified application of IRT models. Two item pairs of the distress scale were expected to be problematic (violating the assumption of local independence) due to the fact that the items of the first pair both refer to sleeping problems and items of the second pair both to residual effects of traumatic experiences. We decided to remove the item of each pair that was lower in discriminative power from further analyses.

In Table 2, the tests of item model fit for GP clients are displayed. Items 17, 22, and 37 showed misfit according to a strict *p* < .01 criterion. Note that the total sample size is large, so these tests are very powerful in detecting slight deviations from the postulated models. In order to get a better view on how ‘bad’ things actually were, Table 3 provides expected (model-based) and observed score frequencies in each category for item 22 (*Listlessness),* which was most problematic according to the χ2 test result. Differences larger than 10 are displayed bold. The last two columns provide observed and expected mean scores for each score level.

**Table 2:** Item-wise *χ* ^2^-tests of model fit for GP-clients (0–2)

Order	Item stem (abbreviated)	*χ* ^2^	*df*	Probability
17	Feeling down or depressed	111.05	45	0.0001
19	Worry	52.67	41	0.1044
20	Disturbed Sleep	68.73	51	0.0494
22	Listlessness	120.55	44	0.0001
25	Tense	47.44	42	0.2598
26	Easily irritated	45.89	47	0.5194
29	That you just can’t do anything anymore	56.45	36	0.0162
31	(…) take any interest in the people and things around you	37.08	38	0.5130
32	That you can’t cope anymore	25.41	37	0.9254
36	That you can’t face it anymore	46.59	33	0.0585
37	No longer feel like doing anything	79.1	35	0.0001
38	Have difficulty in thinking clearly	65.52	45	0.0244
41	Did you easily become emotional	59.84	48	0.1171
48	(…) to put aside thoughts about any upsetting event(s)	67.19	49	0.0431

**Table 3:** Observed and expected score frequencies and mean item scores for different score levels, Item 22, GP-clients, (0–2)

	Cat. 0		Cat. 1		Cat. 2			
Rest score level	Obs.	Exp.	Obs.	Exp.	Obs.	Exp.	M(Obs.)	M(Exp.)
0–7	117	118	41	39	12	14	0.38	0.39
8–16	84	89	120	105	103	112	1.06	1.08
17–20	13	15	44	45	117	114	1.60	1.57
21–23	3	4	12	24	132	118	1.88	1.78
24–25	10	2	14	16	195	201	1.84	1.91
26	0	0	1	4	79	82	1.99	1.95

As can be seen from Table 3, the estimated item parameters for item 22 mimic the response behavior of GP clients quite well: For some cells, observed and expected score frequencies differ somewhat, but mean observed and expected item scores for each score level are always quite close to one another.

We only briefly summarize the most important findings with respect to local independence. Item 20, *Disturbed sleep,* had moderate LD (*χ* ^2^ = 7.5) with the other items of the distress scale. The *χ* ^2^-values for all other items did not exceed 5, and most were even smaller than three. Because the standardized *χ* ^2^-tests for local dependence is only approximately standardized [16], most researchers consider only values greater than ten as indicating relevant local dependence.

Because even for the ‘worst’ fitting item according to significance testing, the differences between observed and expected item score frequencies are not large, combined with the fact that the item parameters model the covariance among items appropriately, we decided that the GPCM is appropriate to represent the response behavior of GP clients.

### IRT-analyses: EHealth clients

The table with item-wise χ2-tests of model fit in the group of eHealth clients can be found in the (Additional file 1: Table A2); here we summarize the most important findings. Again, for three items (17, 25, 29), the *χ* ^2^-test indicated misfit (*p* < .01), of which only item 17 (*Feeling down or depressed)* also showed misfit in the group of GP clients. Comparing observed and expected item scores for items 25 (*Tense*) and 29 (*Just can’t do it anymore*) did not show large discrepancies. For item 17, the observed and expected mean scores for each score level are similar (Additional file 1: Table A3); however, for the lowest score level (0–14), observed and expected responses differed more substantially.

Again, we only briefly report the most important findings with respect to LD: Two items showed moderate LD with the other items: Item 17 (*Feeling down or depressed*; which also was most problematic in terms of model fit; *χ* ^2^ = 7.5) and item 20 (*Disturbed Sleep*; *χ* ^2^ = 7.3). Again, the *χ* ^2^-values for all other items did not exceed five, and most were even smaller than three indicating that the model accounted for most covariance among all item pairs. Thus, also with respect to eHealth clients, we again conclude that the chosen model describes the data quite well.

### Differential item functioning (DIF)

Only two DIF-tests were significant (*p* < .001).^2^ The discrimination parameter (α) of item 38 (*Having difficulty in thinking clearly*; *χ* ^2^ =18.1, df = 1) was higher for eHealth clients (α = 2.15) than for GP clients (α = 1.28). So, item 38 was somewhat more informative for scaling eHealth clients than for scaling GP-clients. The DIF-tests for the interception parameters of Item 17 (*Feeling down or depressed)* was significant (17, *χ* ^2^=12.2, df = 2), indicating that the lowest and highest response categories were relatively more popular among eHealth clients (d_01_ = −.59, d_12_ = −.22) than among GP-clients (d_01_ = −.42, d_12_ = −.06). Out of 42 parameters (14*3), only four differed between GP-clients and eHealth clients. So with respect to structural equivalence, we conclude that this assumption holds for most of the distress items.

In order to evaluate the impact of the differences we found at the scale level, we compared the TCCs of both groups (Fig. 2). Because only three (discriminative power item 38 and interception parameters item 17) out of 42 item parameters differed between groups, we did not expect substantial differences between the TCCs of both groups.

**Fig. 1:**
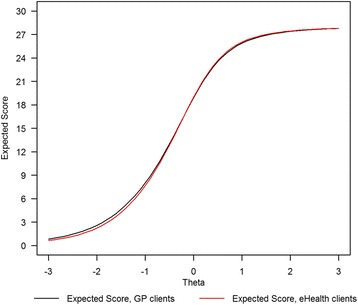
Test characteristic curves distress scale 4DSQ for GP-clients and eHealth clients

Figure 1 confirms our expectation: the two graphs are nearly identical. In fact, it is difficult to discriminate between black and red line. The maximum difference in expected scale scores emerges at θ = −1.5, where the expected scale score of GP-clients is .12 points higher than that of eHealth clients. Because the combined effect of all DIF-effects is negligible at the scale level, the assumption of scalar equivalence holds, and we can use the same cutoff values in both groups for classifying clients as having low, medium, and high levels of distress.

In order to link the cutoff scores (S_x_ ≤ 10 = low, 11 ≤ S_x_ ≤ 20 = medium, and S_x_ ≥ 21 = high) of the total score metric to the IRT-metric, we applied equipercentile linking [17] as follows: We took the GP-sample as reference, because this was the primary group for which the instrument was developed. In the sample of GP clients, 19.8% had a total score of ten or lower, and this total score (S_x_ = 10) corresponds to a theta value of −.82. 47.4% of GP clients had a total score lower than 21, and this total score corresponds to a theta value of −.10. Because scalar equivalence holds, these theta values can be used as cutoff scores ***for classifying clients of both groups*** *:*

- *C*_*low*/*mediun*_ = *θ* <  − . 82.
- *C*_*medium*/*high*_ = *θ* <  − . 10

Note that, according to these cut-off values, approximately 50% of all GP clients and 70% of all eHealth clients report experiencing high levels of distress.

**Fig. 2:**
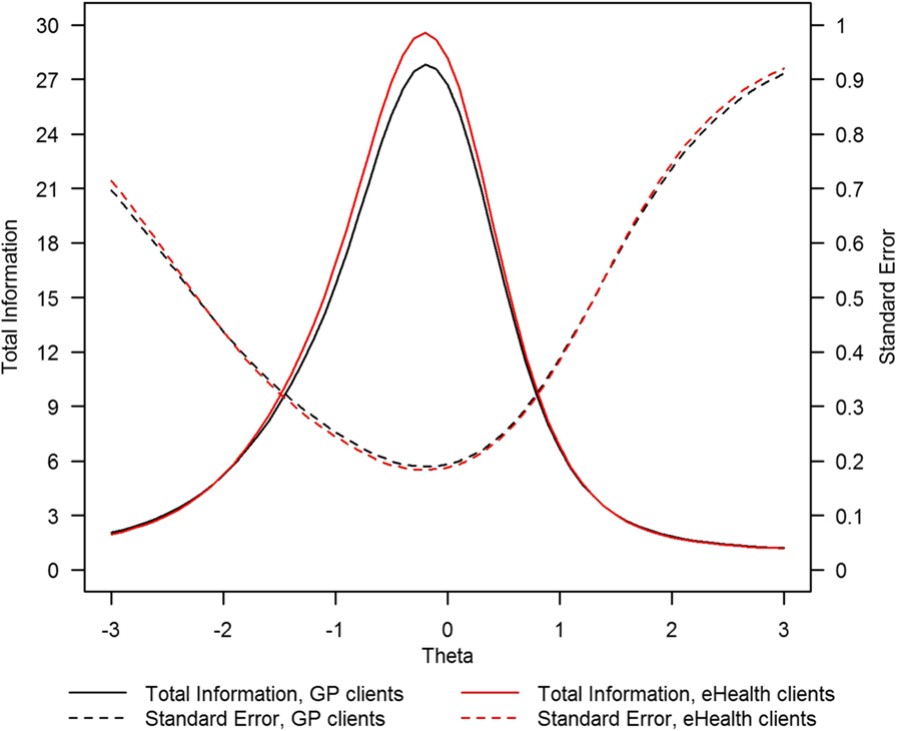
Test information functions and corresponding standard errors for both groups

Figure 2 shows the Test Information Functions (TIFs) and corresponding standard errors for both groups. Recall from the DIF-analyses that only three item parameters differed between groups, so we did not expect to see substantial differences between the TIFs of both groups. Because item 38 provides more information in the sample of eHealth clients, the total information for eHealth clients (red line) is somewhat higher around the mean theta-value of GP clients than the information for GP clients (black line). Measurement precision of the items peaks around the mean value (*θ*_*i*_ = .00) of GP-clients, and is much lower for extreme values. Specifically for high scores (*θ*_*i*_ > .2.00), the estimated standard errors are four times as high as those around the mean value of GP clients. Although the authors of this paper generally strongly favor using standard errors that are conditional on the position of the latent continuum, for convenience, we also provide marginal reliabilities that attempt to sum up the information provided in Fig. 2: Because (i) the spread in levels of distress was lower for eHealth clients than for GP-clients, and (ii) the distress items provided less information for high scoring individuals, the marginal reliability for GP clients (r_xx_ = .89) is somewhat higher than that for eHealth clients (r_xx_ = .83).

Note that measurement precision is very high around the two cutoff scores that were derived earlier for classifying respondents as having moderate (−.82 < θ < −.10) and high (θ > −.10) levels of distress. Because the TCCs of both groups were nearly identical, we can conclude that eHealth clients do experience higher levels of distress than GP clients (M_eHealth_ = .39 & M_GP_ = .00), and that the distress levels of GP clients are more heterogeneous than the distress levels of eHealth clients (SD_eHealth_ = .76, SD_GP_ = 1.00).

### Summary

In general, the commonly estimated item parameters model the response behavior of both GP-clients and eHealth clients quite well. The item that showed some degree of misfit in both groups was item 17, *Feeling down or depressed*. But even for this item, model fit was reasonably good in both groups. Also, the combined effect of all DIF-effects at the scale level, although statistically significant, was found to be negligible. That is, equal total scores represent the same levels of distress in both groups and measurement precision is approximately equal for equal levels of distress in both groups.

## Discussion

### Main findings

The focus of this study was on the generalizability of 4DSQ distress scores across the two samples of GP clients and eHealth clients. We found that the scale measures the same construct in both groups (structural equivalence) and that scale scores in both groups reflect the same levels of distress in both groups (scalar equivalence). Thus, (i) total scores can be used to compare individuals of both groups in terms of their levels of distress, and (ii) the use of equal cutoff scores for classifying members of both groups as having low, medium, and high levels of distress is appropriate. EHealth clients experience higher levels of distress than GP-clients, but the variation in distress scores is less for eHealth clients than for GP-clients. Furthermore, measurement precision of the 4DSQ distress scale is good (SE < .32, say, r_xx_ > .90) for most levels of distress (−1.5 < θ < 1.00), and poor only for levels of distress that are extremely high (*θ*_*i*_>.2.00).

In a recent article [10], a bifactor model was proposed as an appropriate representation for the distress scale. To some readers, this finding may seem incompatible with the use of a unidimensional IRT model. We argue that this is not the case, because (i) the general factor in the bifactor model accounted for more than 95% of the common variance among items, and (ii) the group factor was used by Terluin et al. to model residual covariance among item pairs. Hence, the IRT model that we used and the bifactor model presented by Terluin et al. are very similar.

### Strengths, limitations and future research

One strength of this study was that by means of MGIRT, we were able to derive item parameter estimates based on the data of both groups combined, while fit could still be assessed in both groups separately. Furthermore, we hope that this article encourages clinical practitioners and researchers applying tests and questionnaires in practice to follow the MGIRT approach we used in this article to ensure that their instruments possess the properties of structural equivalence and scalar equivalence in cases where these properties are required.

This study has also limitations. The most prominent one was that we had to remove two out of 16 items prior to analyses because of local dependencies among item pairs. So, the question is whether we may generalize our findings about equivalence to the whole scale (consisting of 16 items). However, because the items that had to be removed correlated very highly with the other item of the pair (*r* = .80–.90), we argue that little item-specific information is lost by removing these two items.

Also, the two samples differed in terms of setting (intake procedure at GP practices versus intake procedure at an EHealth provider) ***and*** medium (paper & pencil versus online). In case we would have found substantial differences at the scale level, as expressed by either differing TCCs or TIFs between the two samples, we would have been unable to attribute these effects to either of these factors. Furthermore, it should also be noted that, because the current study was not a randomized controlled trial, we cannot exclude the possibility that factors that were not incorporated in the study caused the differences we found in mean-levels of distress between groups, or the differences we found in spread between groups, at least to a certain degree.

It should also be noted that for the item that showed misfit in both groups (item 17, *Feeling down or depressed*), the Dutch and English version diverged somewhat. The term used in the original (Dutch) version is ‘*neerslachtigheid’*, for which the best translation would probably be *dysphoria.* This word is not frequently used in English, so probably many respondents would not be familiar with it, which explains the choice of the author for an alternative formulation of this item for the English version. A tentative explanation for item misfit in both groups is that individuals that experience high levels of depression respond differently to ‘dysphoria’ than individuals that experience low levels of depression. High-scoring individuals are perhaps already more used to their level of depression and because of that, more willing to agree with the content than low-scoring individuals, who might find the term ‘too heavy’. However, this is only hypothetical and further research may provide an answer to this hypothesis.

A final limitation is that we were unable to control for the possibility of a constant bias across all distress items. That is, in case eHealth clients overreport the frequency of all symptom experiences the same way across all items, DIF-tests are insensitive to this kind of bias^5^. In order to check the hypotheses of such a structural reporting bias, objective information on the distress-status (diagnosis of burnout and sick-leave for example) of respondents in both groups would be required.

## Conclusions

The distress items of the 4 DSQ have largely the same meaning for GP patients and eHealth clients. Similar total scores reflect similar levels of distress in both populations, and thus the use of similar cut-off scores for classifying respondents as having low, medium and high levels of distress can be defended.

## Additional file

Additional file 1: a) **Table A1.** Distress items of the Four-Dimensional symptom questionnaire (4DSQ). b) **Table A2.** Item-wise Χ^2^-tests of model fit for eHealth clients. c) **Table A3.** Observed and expected score frequencies and mean item scores for each score level Item 17, eHealth clients. d) Technical information on the GPCM. In this section, we provide further technical information on the Generalized Partial Credit Model, the Item Response Theory Model that we used to analyze the data. e) **Figure A1.** Category response curves for Item 22. f) Technical details DIF tests. In this section, we provide technical details of the Differential Item Functioning tests that we used in our study for the interested reader. g) Full information on MGIRT analyses. In this section, we provide a more detailed description of Multiple Group Item Response Theory analyses, which we used to analyze the datasets. (DOCX 29 kb)

## Abbreviations

4DSQ: Four Dimensional Symptom Questionnaire
DIF: Differential Item Functioning
DTF: Differential Test Functioning
GP: General Practitioner
GPCM: Generalized Partial Credit Model
IRT: Item Response Theory
LD: Local Dependence
LSI: Local Stochastic Independence (
MGIRT: Multiple Group Item Response Theory
TCC: Test Characteristic Curve
TIF: Test Information Function

## Footnotes

1 Interapy® originated from the University of Amsterdam. It is a certified provider for primary- and specialized mental health care, with a special interest in research. For more than 10 years, the organization has been offering evidence-based eHealth interventions for various mental health disorders. Only secured/protected websites are used for the contact between coach/therapist and health care recipient.

2 A detailed description of the procedure we used to test items for DIF can be found in the Additional file 1 in the section *Technical details DIF tests*.
