# Supplementary material for: Identifying levels of general distress in first line mental health services: can GP- and eHealth clients’ scores be meaningfully compared?
Source: BMC Psychiatry. 2017 Dec 1;17:382. doi: 10.1186/s12888-017-1552-3 (PMC5709985; doi:10.1186/s12888-017-1552-3)
Supplement: Additional file 1: — a) Table A1. Distress items of the Four-Dimensional symptom questionnaire (4DSQ). b) Table A2. Item-wise Χ2-tests of model fit for eHealth clients. c) Table A3. Observed and expected score frequencies and mean item scores for each score level Item 17, eHealth clients. d) Technical information on the GPCM. In this section, we provide further technical information on the Generalized Partial Credit Model, the Item Response Theory Model that we used to analyze the data. e) Figure A1. Category response curves for Item 22. f) Technical details DIF tests. In this section, we provide technical details of the Differential Item Functioning tests that we used in our study for the interested reader. g) Full information on MGIRT analyses. In this section, we provide a more detailed description of Multiple Group Item Response Theory analyses, which we used to analyze the datasets. (DOCX 29 kb) [file 12888_2017_1552_MOESM1_ESM.docx]

**Additional file**

**Table A1.** Distress items of the Four-Dimensional symptom questionnaire (4DSQ)

| **Order** | **Item** |
| --- | --- |
| 17 | During the past week, did you suffer from feeling down or depressed? |
| 19 | During the past week, did you suffer from worry? |
| 20 | During the past week, did you suffer from disturbed sleep? |
| 22 | During the past week, did you suffer from listlessness? |
| 25 | During the past week, did you feel tense? |
| 26 | During the past week, did you feel easily irritated? |
| 29 | During the past week, did you feel that you just can’t do anything anymore? |
| 31 | During the past week, did you feel that you can no longer take any interest in the people and things around you? |
| 32 | During the past week, did you feel that you can’t cope anymore? |
| 36 | During the past week, did you feel that you can’t face it anymore? |
| 37 | During the past week, did you no longer feel like doing anything? |
| 38 | During the past week, did you have difficulty in thinking clearly? |
| 39 | During the past week, did you have difficulty in getting to sleep? |
| 41 | During the past week, did you easily become emotional? |
| 47 | During the past week, did you ever have fleeting images of any upsetting event(s) that you have experienced? |
| 48 | During the past week, did you ever have to do your best to put aside thoughts about any upsetting event(s)? |

**Table A2.** Item-wise $\chi^{2}$-tests of model fit for eHealth clients.

| **Order** | **Item stem** | $\boldsymbol{\chi}^{\boldsymbol{2}}$ | **df** | **Probability** |
| --- | --- | --- | --- | --- |
| 17 | Feeling down or depressed | 115.21 | 43 | 0.0001 |
| 19 | Worry | 36.84 | 41 | 0.6568 |
| 20 | Disturbed Sleep | 70.2 | 47 | 0.0157 |
| 22 | Listlessness | 54.85 | 41 | 0.0725 |
| 25 | Tense | 69.92 | 42 | 0.0044 |
| 26 | Easily irritated | 56.44 | 45 | 0.1176 |
| 29 | That you just can’t do anything anymore | 62.24 | 36 | 0.0043 |
| 31 | (…) take any interest in the people and things around you | 41.97 | 36 | 0.2273 |
| 32 | That you can’t cope anymore | 28.6 | 34 | 0.7303 |
| 36 | That you can’t face it anymore | 51.16 | 34 | 0.0296 |
| 37 | No longer feel like doing anything | 20.74 | 34 | 0.9641 |
| 38 | Have difficulty in thinking clearly | 44.53 | 42 | 0.3650 |
| 41 | Did you easily become emotional | 39.89 | 45 | 0.6885 |
| 48 | (…) to put aside thoughts about any upsetting event(s) | 45.99 | 44 | 0.3912 |

**Table A3.** Observed and expected score frequencies and mean item scores for each score level Item 17, eHealth clients.

|  | **Cat. 0** | | **Cat. 1** | | **Cat. 2** | |  |  |
| --- | --- | --- | --- | --- | --- | --- | --- | --- |
| **Rest score level** | **Obs.** | **Exp.** | **Obs.** | **Exp.** | **Obs.** | **Exp.** | **M(Obs.)*** | **M(Exp.)*** |
| **0-14** | ***69*** | ***104*** | ***146*** | ***97*** | ***64*** | ***78*** | 0.98 | 0.91 |
| **15-18** | ***20*** | ***31*** | 78 | 75 | 137 | 129 | 1.50 | 1.42 |
| **19-20** | 13 | 10 | ***25*** | ***39*** | ***119*** | ***108*** | 1.68 | 1.62 |
| **21-22** | 12 | 7 | ***23*** | ***39*** | ***172*** | ***161*** | 1.77 | 1.74 |
| **23-24** | 7 | 4 | ***20*** | ***31*** | 227 | 219 | 1.87 | 1.85 |
| **25** | 4 | 1 | 7 | 10 | 115 | 114 | 1.88 | 1.90 |
| **26** | 0 | 0 | 4 | 9 | 147 | 143 | 1.97 | 1.94 |

* cat0 = 1, cat1 = 2 & cat2 = 3.

*Technical information on the GPCM*

From these specifications, so-called category response curves can be deduced. In Figure 1, the category response curves for Item 22 (recoded into three response options) are plotted. As can be seen, with increasing trait level, the probability of choosing the lowest response category becomes less likely and the probability of choosing the higher response options becomes more likely. At each point on the latent continuum, these category response probabilities sum up to one. For example, a person who is situated at approximately $\theta i$=-.70, a response in the lowest category is equally likely as a response in the second response category, and the highest response category is very unlikely. Item *j* displayed in Figure 1 has three response options and is characterized by the following set of intersection parameters: b_j1_=-.70, b_j2_=-.40.

**Figure A1.** Category response curves for Item 22

*Technical details DIF tests*

To test specific items for possible DIF, an *anchor* is needed, consisting of items that define the trait reasonably well and that are known to be unbiased. These items may be chosen based on previous research findings or theoretical considerations. In case no such previous knowledge exists, all other items of the scale serve as a preliminary anchor when an item is tested for DIF. In either case, testing for DIF is an iterative purification procedure (Lord, 1980): step by step, items that exhibit DIF are removed from the anchor and items that do not exhibit DIF are added to the anchor. DIF-tests essentially evaluate whether the increase in fit by freeing parameter estimates between groups is worth the number of additional parameters that have to be estimated. Specifically, two models are compared. The model were item parameter estimates are constrained to be equal between groups is called *compact model* and the model were item parameters are freely estimated within each group is called *augmented model*. Under the null hypothesis of no DIF, the difference between -2* the log-likelihood of each models follows a chi-square distribution with degrees of freedom equal to the number of additional parameters.

*Full information on MGIRT analyses*

Specifically, we assessed item-wise model fit in each group by means of sum score based $\chi^{2}$-statistics (S-X^2^) that compare the frequencies of expected (model-based) category score frequencies to observed category score frequencies. Because these test statistics are very sensitive with large samples, we inspected the differences between observed and expected category score frequencies for different score levels (i.e., the total score without the item targeted) for those items that showed the worst fit (p<.01). Instead of doing this for each score level, we collapsed score levels in such a way as to create expected category score frequencies of at least one hundred persons in each cell. Additionally, to check the magnitude of possible LD among item pairs, we computed marginal $\chi^{2}$-statistics to test whether the residual correlations for each item with all other items are actually close to zero. Because these $\chi^{2}$-statistics are only approximately standardized, we followed the recommendation of Chen and Thissen (1997) to consider values larger than 10 as large, indicating likely LD, and values between 5 and 10 as indication of moderate LD.
